# Improved Infrared-Sensing Running Wheel Systems with an Effective Exercise Activity Indicator

**DOI:** 10.1371/journal.pone.0122394

**Published:** 2015-04-13

**Authors:** Chi-Chun Chen, Ming-Wen Chang, Ching-Ping Chang, Wen-Ying Chang, Shin-Chieh Chang, Mao-Tsun Lin, Chin-Lung Yang

**Affiliations:** 1 Department of Electronic Engineering, National Chin-Yi University of Technology, Taichung, Taiwan; 2 Department of Electrical Engineering, Southern Taiwan University of Science and Technology, Tainan, Taiwan; 3 Department of Biotechnology, Southern Taiwan University of Science and Technology, Tainan, Taiwan; 4 Department of Electrical Engineering, National Cheng Kung University, Tainan, Taiwan; 5 Department of Medical Research, ChiMei Medical Center, Tainan, Taiwan; University of Rome Foro Italico, ITALY

## Abstract

This paper describes an infrared-sensing running wheel (ISRW) system for the quantitative measurement of effective exercise activity in rats. The ISRW system provides superior exercise training compared with commercially available traditional animal running platforms. Four infrared (IR) light-emitting diode/detector pairs embedded around the rim of the wheel detect the rat’s real-time position; the acrylic wheel has a diameter of 55 cm and a thickness of 15 cm, that is, it is larger and thicker than traditional exercise wheels, and it is equipped with a rubber track. The acrylic wheel hangs virtually frictionless, and a DC motor with an axially mounted rubber wheel, which has a diameter of 10 cm, drives the acrylic wheel from the outer edge. The system can automatically train rats to run persistently. The proposed system can determine effective exercise activity (EEA), with the IR sensors (which are connected to a conventional PC) recording the rat exercise behavior. A prototype of the system was verified by a hospital research group performing ischemic stroke experiments on rats by considering middle cerebral artery occlusion. The experimental data demonstrated that the proposed system provides greater neuroprotection in an animal stroke model compared with a conventional treadmill and a motorized running wheel for a given exercise intensity. The quantitative exercise effectiveness indicator showed a 92% correlation between an increase in the EEA and a decrease in the infarct volume. This indicator can be used as a noninvasive and objective reference in clinical animal exercise experiments.

## Introduction

Recent studies have shown that regular exercise generates new neurons, increases the volume of brain structures, and improves the functional cognition, in both humans and higher animals [1–7]. This has been shown on both treadmill and running wheel platforms [4][8–11]. Clinical research [12][13] and animal studies [7][14][15] have presented evidence suggesting that regular physical activity may confer neurophysiological benefits in people with medical conditions such as Parkinson’s disease [16], ischemic stroke [17], depression [18], metabolic syndrome [19], and Alzheimer’s disease [20]. Neurophysiological mechanisms resulting from exercise in humans are similar to those in animals. Accordingly, animal models are often used to help verify the efficacy of exercise as a preventative therapy in human beings [11][21–25]. Experiments verifying the efficacy of exercise use rodents trained on a treadmill or a running wheel. Training involving forced exercise compels a rodent to run at a fixed velocity and for a fixed duration. The training intensity is generally calculated on the basis of the exercise duration at a certain speed [11][21][22][24]. This is the current method for estimating the amount of exercise required for neurophysiological protection. However, sometimes the exercise is ineffective when the rodent stumbles and falls or, alternately, is reluctant to run [26]. Ineffective exercise has reduced or no benefits. While there are no accepted approaches to quantify effective exercise, devising a method for quantifying effective exercise would enhance the utility of rodent exercise studies. Thus, this study presents a novel microprocessor-controlled forced running wheel system with the following advantages and features: The infrared-sensing running wheel (ISRW) system 1) records the exercise activity of a test rodent in a hard disk, 2) employs a quantitative indicator of effective exercise for use in clinical animal exercise experiments, and 3) automatically executes training patterns to teach rats to run steadily for up to an hour at 30 m/min, by using training patterns. The ISRW system was successfully used as an animal stroke prevention model by a hospital group performing middle cerebral artery occlusion (MCAo) experiments, and it was found to be considerably superior to existing systems.

Current commercially available platforms, such as treadmills and passive or motorized running wheels, have limitations [27]. Treadmills use electric shocks to force rats to run, introducing the effect of psychological stress in the experimental results [24][28][29]. There are two types of running wheels: one is a motorized running wheel (MRW) and the other is a voluntary running wheel (VRW). In the MRW, a motor is used to drive the running wheel, forcing rats to run at a specific speed. The VRW is an un-motorized wheel that allows spontaneous running. While both these running wheels produce less psychological stress than a treadmill [8][28–31], they have limitations. VRW devices used in spontaneous running systems are significantly affected by the ability of rodents to vary the exercise intensity [32]. With commercially available MRWs, experiments have shown that rodents cannot run at speeds exceeding 20 m/min [26][33–34]. However, specific clinical endpoints have shown that high-intensity exercise (exceeding 20 m/min) produces better neurophysiological protection and recovery [9][10][35]. In earlier studies, high-intensity exercise or variable speed movement was forced upon rats by using a conventional MRW runway [26][36]. This worked adequately, but some rats showed a reluctance to run, which significantly reduced the effective exercise.

The ISRW platform proposed in the current study is intended to minimize interference factors while providing reliable and quantifiable training for different running intensities. The ISRW platform overcomes the speed limit problem faced in commercial MRW training. The ISRW system was used for the prevention of ischemic strokes in rodents by a hospital group associated with this study. Moreover, the ISRW system enables the exercise difficulties of animals with different motor abilities, such as rat models of neurological spinal injury and Parkinson deficits, to be considered. Importantly, the speed and training regime can be modified for according to rat capability or exercise intensity levels. The design considerations and features of the proposed ISRW system are presented in the next (Materials and Methods) section. An animal ischemic stroke model is used to validate the system’s effectiveness. The ISRW experimental results are compared with the results of treadmills and MRW systems. The ISRW system is equipped with infrared transmitter/detector modules mounted near the wheel’s rim for monitoring the position of the rats. In exercise-related stroke prevention experiments, a quantitative indicator of the overall exercise effectiveness (*QEI*_*ISRW*_) is defined and calculated. The indicator serves as a noninvasive and objective reference.

## Materials and Methods

The ISRW system is built around a conventional microcontroller, and it can automatically measure the effective exercise activity of laboratory animals such as rats. In addition, it can automatically train rats to run at high speeds (approximately 30 m/min for an hour).

### Infrared-sensing running wheel system

The ISRW system consists of five parts: (a) a large-sized transparent acrylic running wheel with a polyvinyl chloride (PVC) track, (b) a rubber wheel (diameter: 10 cm) mounted on a brushless DC electric motor that drives the running wheel through friction, (c) a microcontroller that controls the motor speed according to an automatic progressive training pattern, which is useful for training rats to run at high speeds, (d) four infrared (IR) light-emitting diode (LED)/detector pairs just outside the running wheel for detecting the rat’s real-time position, and (e) a conventional PC downloads firmware for the microcontroller. The firmware controls the rotational speed of the wheel and IR detection of the rat’s position. The PC downloads the data to the microcontroller via a parallel port, while a serial RS232 link monitors the test data and saves the data to a hard drive.

### Apparatus

Two MRWs were constructed for the present study. One was a traditional MRW for use as the baseline, and the other was the proposed ISRW device. The MRW wheel was built in our laboratory from a commercial un-motorized wheel (Large Free Wheel, Reid Pet Stores, Taiwan). The constructed MRW was 12 cm wide and 35 cm in diameter, i.e., typical of current commercial un-motorized running wheels. A DC electric motor (BLEM512-GFS, Oriental Motor, Japan) is positioned at the central axis by using the 1/1 gearing arrangement typical of traditional MRWs. It is noteworthy that rats in our experiments were reluctant to run at 20 m/min or higher speeds on the bars of the conventional MRW runway structure. Rats attempted to grab the bars of the runway when they could not keep up with the high speed of the MRW. For training the MRW group of rats for a comparison with other high-speed platforms, a PVC belt was used to cover the outside of the floor of the MRW. This arrangement left bars on the floor of the wheel, which rats sometimes caught hold of, but the design of our MRW reduced the number of catching events considerably and enabled rats to run at 30 m/min.

**Fig 1:**
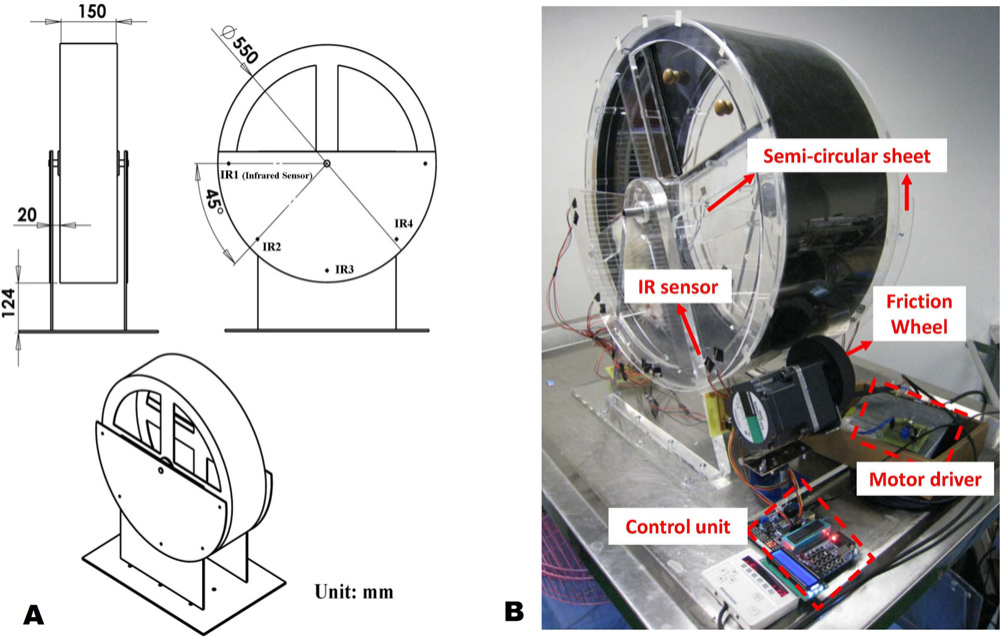
ISRW system apparatus. (a) Design drawing. (b) Actual photograph. The IR1–IR4 represents the locations of infrared sensing pairs.

The ISRW was built around a custom-designed seamless acrylic wheel with a width of 15 cm and a diameter of 55 cm (Fig 1(a)); in other words, it was wider and had a larger diameter compared with the MRW. A photograph of the lab-built ISRW apparatus is shown in Fig 1(b). The lower part curvature of the larger ISRW wheel allowed a rat more response time for maintaining the training speed and helped prevent the rat from stumbling at high running speeds (>20 m/min). The rotational speed of the ISRW was controlled by friction against the 10-cm diameter rubber wheel on the DC electric motor (BLEM512-GFS, Oriental Motor, Japan) mounted in a transverse position relative to the acrylic wheel’s outer runway. This outer-edge drive methodology provided a large torque for rotating the wheel with high speed and high acceleration control. With a motor torque of 8.6 N·m, the combined system delivered more than 1.2 kg in addition to the 250 to 350 g mass of a rat, providing a sufficient margin for a virtually instant reaction to the force generated by a running rat. The inner runway of the ISRW was covered with a PVC belt, creating a smooth but lightly textured surface that allowed rats to run effectively and helped prevent injuries because of slipping and stumbling.

### Control unit

**Fig 2:**
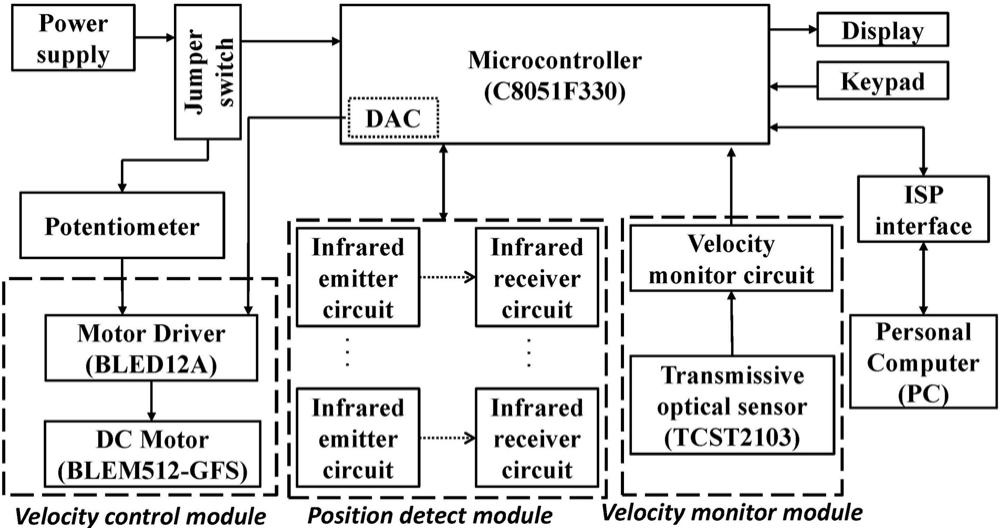
Block diagram of the microcontroller-based control unit.

A block diagram of the control unit is shown in Fig 2. The control unit was built around a C8051F330 microcontroller (Silicon Labs, USA). The manual jumper switch allowed the user to switch between the learning and automatic modes. The input/output interface included a 10-digit liquid-crystal display (LCD) for displaying the instantaneous rotational speed of the wheel and an LED display for providing the rat position; the LED display showed numbers from 1 to 4 for indicating rat positions of 0°, 45°, 90°, and 135°. Fig 2 includes a velocity monitor module, a velocity control module, and a position detection module. The velocity was monitored using an OPX-2A speedometer (Oriental Motor, Japan). The errors between the velocity monitor module and the OPX-2A speedometer were within ± 0.01 m/min. A regulated 5 V power supply provided power to the ISRW control system. The microcontroller had two primary functions: 1) It accepted commands for controlling the wheel speed through the digital-to-analog converter (DAC) by using pulse-width modulation (PWM), and controlled the motor driver according to the commands. 2) It obtained the instantaneous location of a rat exercising on the running wheel on the basis of data received from the IR transmitter/receiver pairs located near the running wheel.

### Velocity control module and adaptive training model

There were two velocity control modes: learning and automatic modes. The mode was selected using the manual setting of the jumper switch. A new rat was given three days to become familiar with the wheel and to get used to running for an extended period. At the beginning of training, even healthy rats stumbled and fell easily at a speed of 20 m/min. Training was started by a human operator who, using a manual potentiometer, started the ISRW wheel slowly and gradually increased the speed to 20 m/min. If the rat could not maintain the training speed, the speed was reduced and then slowly increased again. This process continued for three days for 30 min of each day’s training session. This was the learning mode and it was used for the first three days.

Automatic mode training began on the fourth day and lasted for three weeks. It provided a consistent and precise running pattern that eliminated human subjective factors. In the automatic mode, the wheel speed slowly increases and reaches the maximum programmed speed in approximately 3 min. There are three automatic training patterns. The first week teaches the rat to run for 30 min at 20 m/min, the second week teaches it to run at 30 m/min for 30 min, and the third week teaches it to run at 30 m/min for 60 min. Training is complete at the end of three weeks. Automatic velocity control of the ISRW is achieved using the microcontroller (C8051F330), a 10-bit DAC, a motor driver (BLED12A, Oriental Motor, Japan), and the DC motor (BLEM512-GFS, Oriental Motor, Japan).

**Fig 3:**
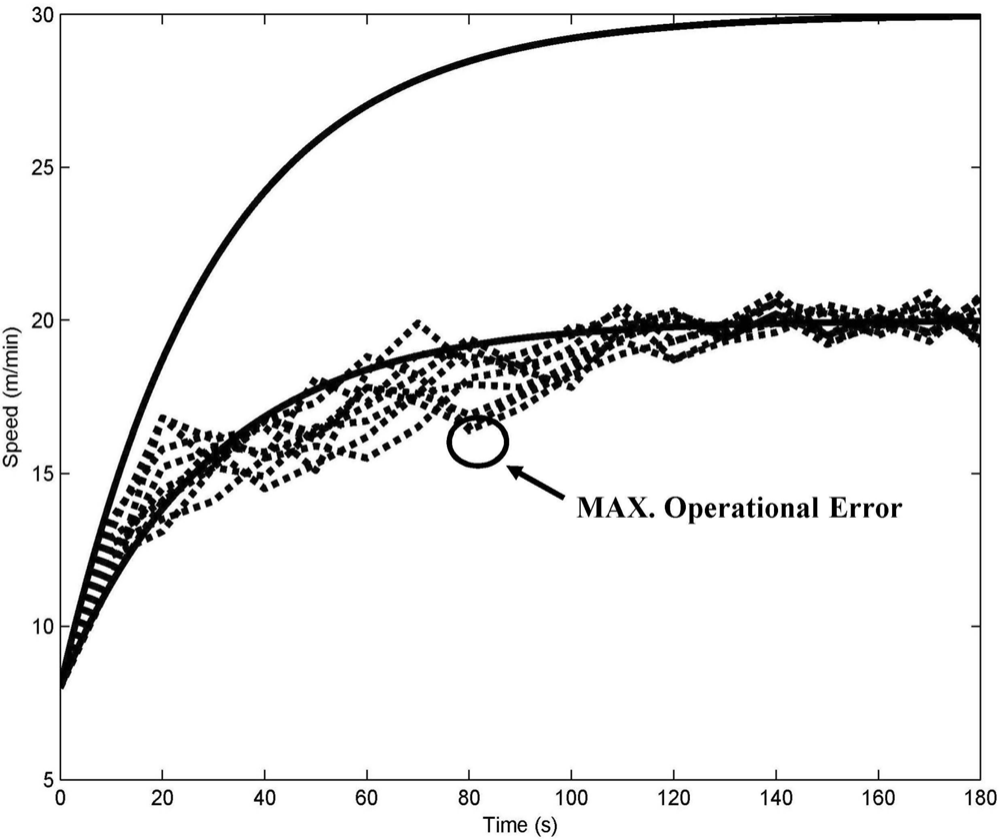
Construction of an adaptive exponential exercise acceleration training model. The dotted lines represent the manual acceleration curves of 7 rats on Day 3. The solid line represents the automatic adaptive acceleration curve. The circle marker indicates the manual maximum operational deviation (14.4%).

Nine rats were used in experiments conducted for constructing the training patterns for the first three days of the learning mode. In Fig 3, the dotted lines represent the acceleration curves of the nine rats on Day 3. The solid line represents the automatic adaptive acceleration curve fit to the trends observed in the learning acceleration training curves of the first three days. The scattered lines imply poor repeatability using manual speed-up control.

The adaptive acceleration training curve of the first week’s automation pattern, which is based on the experimental velocity data, shows an exponential distribution similar to the velocity data distribution. Therefore, an optimal parameter set of an exponential exercise acceleration training curve was constructed (solid line in Fig 3); the x-axis represents time and the y-axis represents the corresponding controlled speed. The speed (*V*_ISRW_) at time *t* can be formulated as

$$V_{ISRW}\left(t\right)=A\;\times \;\left(1\;-\;e^{-t/\tau }\right)\;+\;B$$ (1)

where *A* and *B* are fitted constants and *τ* is the time constant of the increasing speed. After fitting the experimental data for the first and second weeks into the training models, *A* and *B* were determined to be (12 and 8) and (22 and 8), respectively. The acceleration (given by the slope of the velocity curve) was obtained by changing the time constant *τ* (*τ* is set to 30). This consistent acceleration training model based on (1) reduced the maximum operational human error (*V*_*OE*_(*t*)) by 14.4% in our experiments (Fig 3, circle marked). The maximum error in the running distance (*Δs*, 2.12 m) occurred in the initial 3 min and could be calculated by the time integration of learning speed control ($\int V_{1}\left(t\right)$). The proposed control speed ($\int V_{c}\left(t\right)$) has a repeatable and precise pattern and eliminates variations, as shown in (Eq 3). The operational error is calculated as follows:

$$V_{OE}\left(t\right)\text{\% = }\left(\left(V_{c}\left(t\right)\;-\;V_{1}\left(t\right)\right)\;/\;V_{c}\left(t\right)\right)\;\times \;100\%$$ (2)

$$\Delta s\;=\;\int V_{c}\left(t\right)dt\;-\;\int V_{1}\left(t\right)dt$$ (3)

### Position detection module

**Fig 4:**
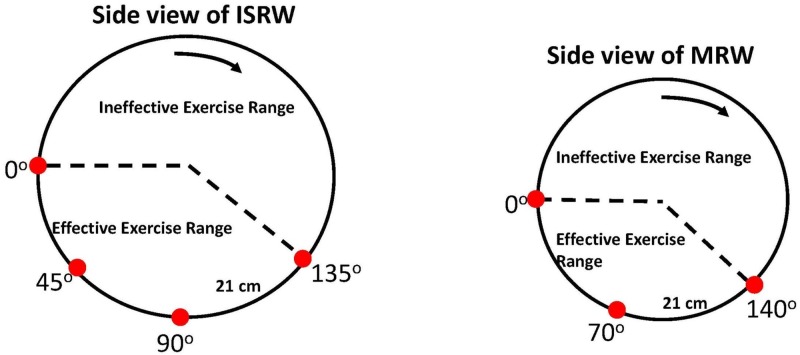
Deployment of the IR sensors.

The ISRW uses IR sensing to monitor a test animal’s position and to observe rat exercise behavior. This information is recorded and used to determine the effective exercise activity of the monitored animal. The four IR LED/detector pairs sense the position of the running rat. Normally, only one IR source/detector pair is triggered at a given moment. If two are triggered, the software chooses one of the two signals randomly. In the ISRW, IR source/detector pairs are located every 45°, approximately 2 cm outside the rim of the running wheel. The arc interval between IR source/detector pairs is 21 cm (shown in Fig 4(a)), which is approximately equal to the length of the test rats. The four IR source/detector pairs are positioned from 0° to 135° (Fig 4(a)), with 90° being defined as the bottom position. Experimental observations of the ISRW showed that when rats exercised normally, they persistently ran and attempted to confine themselves within the region covered by the four IR source/detector pairs. Therefore, this region is designated as the region of effective exercise activity (EEA), which implies normal and effective running. Conversely, the region outside the EEA is designated the region of ineffective exercise activity (IEA). For example, if a running rat stumbles and the wheel continues to move, then the rat is out of control and is dragged into the IEA region. The rat can recover and begin running again later in the EEA, but the rat’s activity in the IEA cannot be considered as effective exercise. Therefore, a quantitative effectiveness indicator (*QEI*_*ISRW*_) is proposed for indicating the percentage of time the rat spends in the EEA region, and it is mathematically expressed as

$$QEI_{ISRW}\%\;=\;EEA/\left(EEA+IEA\right)\;\times \;100\%$$ (4)

The use of IR sensors in the MRW is similar that in the ISRW. Because the MRW (diameter: 35 cm) is smaller than the ISRW, the sensors are deployed from 0° (the left horizontal position) to 140°. An IR source/detector pair (arc interval: 21 cm) is placed every 70°, as shown in Fig 4(b). The region covered by the three IR source/detector pairs is defined as the EEA region.

The IR sensors of the ISRW system monitor the real-time location of a running rat. Moreover, the IR sensors can use the location information and control the motor speed for training animals. For example, when a rat with a low motor speed triggers the IR sensor at 0° and is about to fall, it can be inferred that the rat cannot keep up with the training speed. In this case, the ISRW can be programmed to reduce the speed of the wheel to prevent the rat from falling and being injured. In the ISRW system, an SIR-56ST3F IC (Rohm, Japan) is used as the IR transmitter module and RPM7138 (Rohm, Japan) is used as the IR receiver module. The outputs of the four IR detectors are connected to the P0.4, P0.5, P0.6, and P0.7 input pins of the microcontroller.

### Software

**Fig 5:**
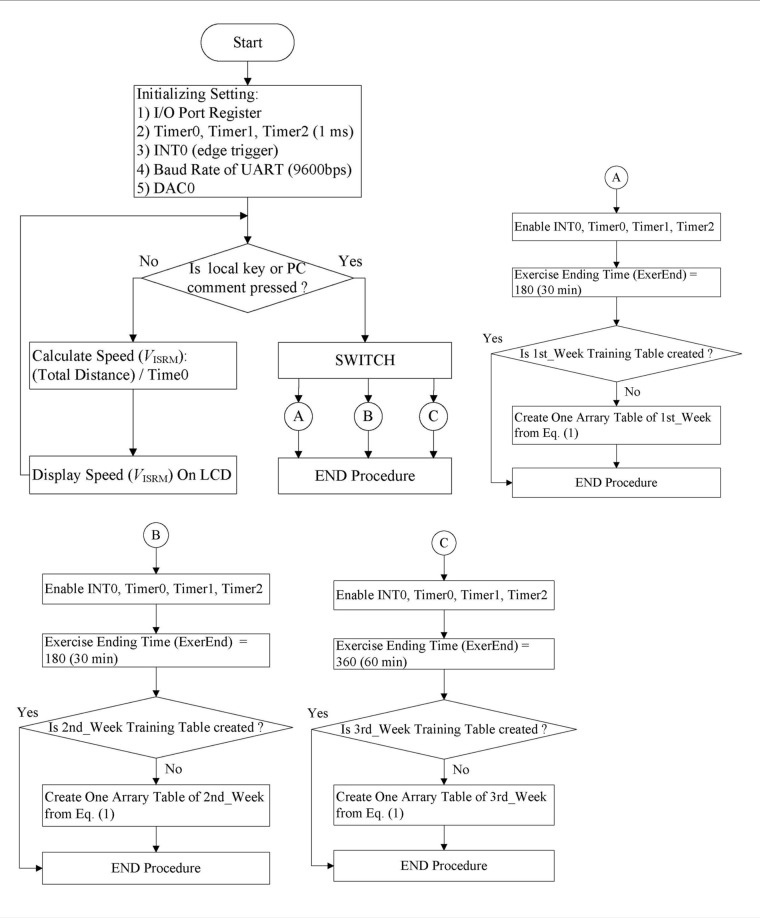
Flowchart of the main microcontroller program.

The software used in the ISRW is divided into two parts: (a) PC software containing instructions for the user interface (UI), weekly training speed, data storage, data display, and data processing, and (b) microcontroller firmware used for controlling the rotational speed of the ISRW for detecting the position of the rat, displaying the position data of the rat on the seven-segment LED, and transferring data to the PC. The microcontroller firmware is written in C language, and it includes the main program, three timer interrupt service routines, and one external interrupt service routine. The flow chart for the entire system is presented in Fig 5. The main program loop calculates and displays the training speed, which is displayed on the LCD. The main program then awaits commands #A–#C, which correspond to the three formal weekly adaptive training curves. These commands may be input from either the keypad or the PC.

**Fig 6:**
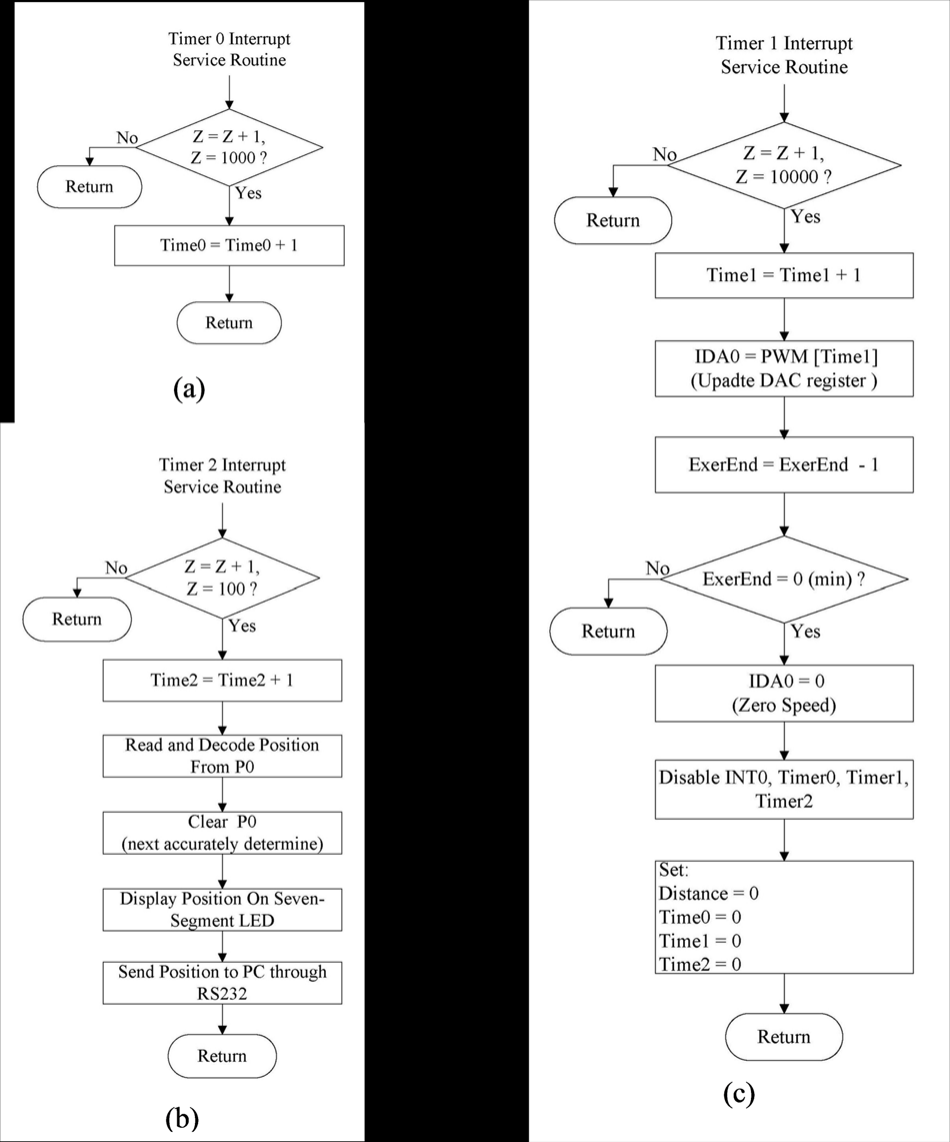
Flowchart of the three timer service routines of the microcontroller.

Three timer interrupt service routines are used: Timer0, Timer1, and Timer2 (Fig 6(a), 6(b), and 6(c), respectively). Timer0 calculates the time (in seconds) taken for a complete wheel rotation. Timer1 generates the PWM value for the time unit, which is used to update the PWM value every 10 s until the end of the exercise (ExerEnd). Timer2 estimates the position every 0.1 s on the basis of the P0 register and sends the data to the PC for storage and analysis. Four IR LED/detector pairs—IR1, IR2, IR3, and IR4—detect the position of the rats during the training period. The output signals of IR1, IR2, IR3, and IR4 are directly sent to the general I/O port P0 of the microcontroller. Ineffective exercise occurs when there is no detector output signal and the IR1–IR4 values in the P0 register are null. The parameter *QEI*_*ISRW*_ is calculated on the PC.

**Fig 7:**
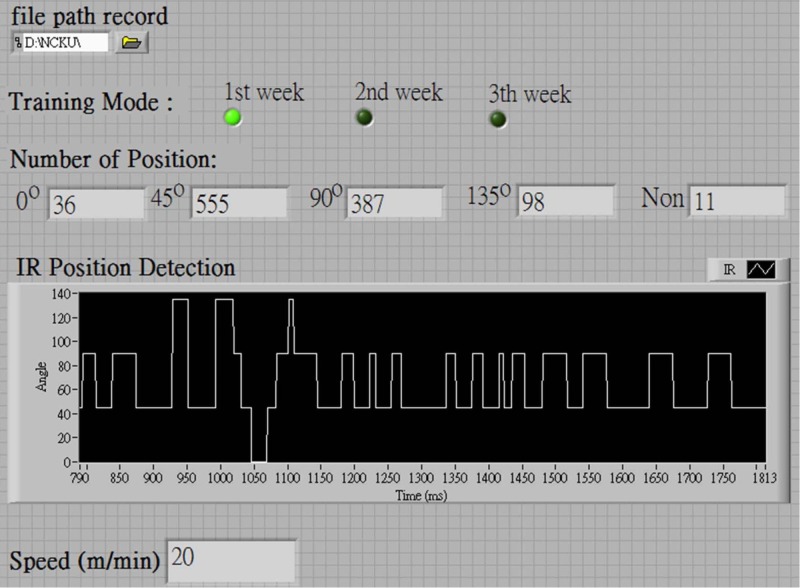
User interface on the PC (LabView program).

The PC program of the platform is written in LabVIEW and includes an RS-232 interface between the PC and the ISRW system control unit. When the weekly training command is given, the LabVIEW program receives the position readings every 0.1 s from IR LED/detector pairs IR1 to IR4. According to the commands #A–#C given to the LabVIEW program, data on the instantaneous position of the rat can be stored on a hard disk and displayed graphically on the local PC monitor, as shown in Fig 7. The above is for the ISRW system, which is the topic of this paper. Details regarding the MRW and treadmill systems are not provided in this paper, to minimize the discussion.

## Testing ISRW System

The following protocol was used to verify the ISRW system operation. Rats were randomly assigned to one of five groups—sham (no exercise, no stroke), control (no exercise, stroke), treadmill (treadmill exercise, stroke), MRW (MRW exercise, stroke), and ISRW (ISRW exercise, stroke)—and there were nine rats per group. After preliminary manual training for three days, the treadmill, MRW, and ISRW exercise groups followed a 3-week exercise training program. To determine the EEA, the positions of the rats in the MRW and ISRW groups were recorded using the developed IR modules. After 3 weeks, a stroke was induced in each rat (excluding the sham group) through 60-min MCAo followed by 7-day reperfusion using an intraluminal filament. The same surgery was performed for the sham group without leading to any blocked arteries. To evaluate the damage caused by and recovery from the stroke, the modified neurological severity score (mNSS) [37] was determined and an inclined plane device test [38] was performed for the next seven days. Subsequently, all animals were sacrificed and exposed to triphenyltetrazolium chloride (TTC) to assess the infarct volume. Finally, *QEI*_*ISRW*_ for the EEA was compared with the infarct volume.

### Calibration

**Fig 8:**
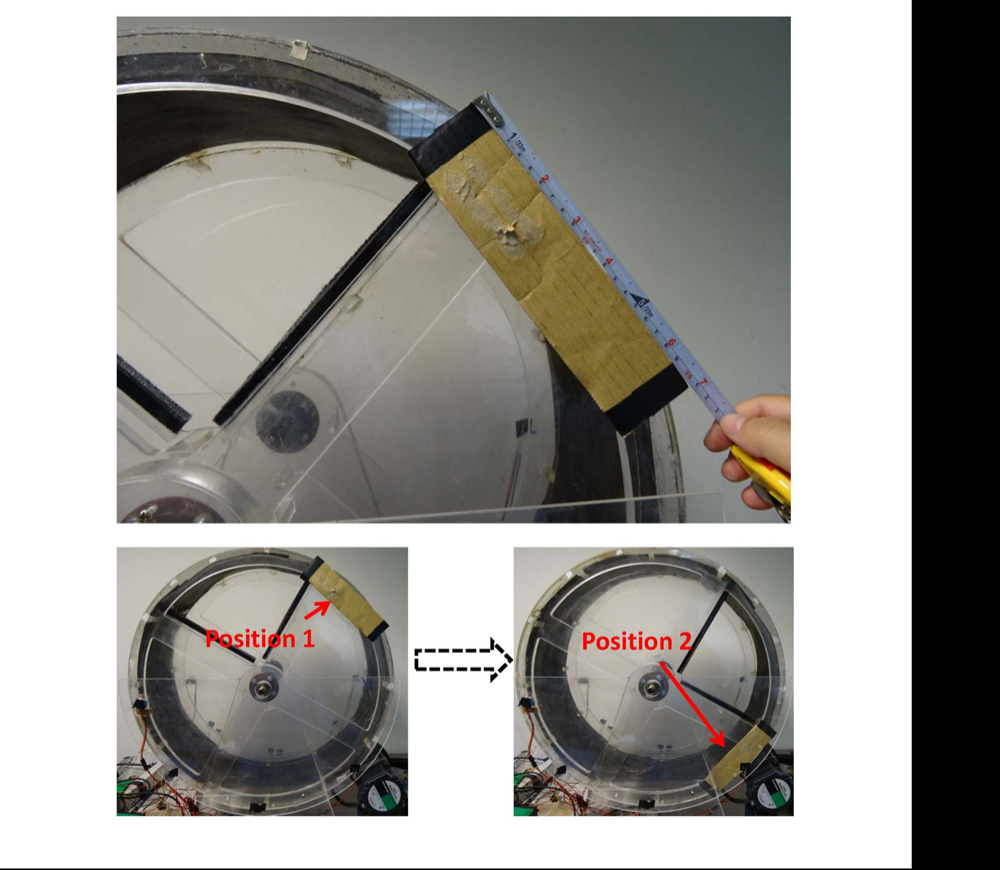
A 20-cm opaque piece of cardboard is used to verify the IR sensing accuracy.

The capability of the ISRW system to locate the position of a running rat depends on the speed of the rotating wheel and the distance between the transmitter and the receiver in a transmitter-receiver pair. The distance between the IR transmitter and the IR receiver for each detection position is fixed at 19 cm. Therefore, only the rotational speed can affect the IR sensing capability and accuracy. To test the position detection limitation of the implemented ISRW system for the highest operational velocity, a 20-cm long opaque piece of cardboard mimicking the length of a rat was stuck to the side of the wheel by using a black tape (Fig 8). The speed of the wheel was increased gradually from 20 m/min (with an increment of 10 m/min for each calibration step) to verify the sensing accuracy of the IR LED/detector pairs. The test results indicated that 100% correct sensing was achieved for speeds in the range 20–100 m/min. When the speed exceeded 110 m/min, the sensing error was 4%. Therefore, sensing for speeds up to 100 m/minute was reliable, and it is concluded that the implemented IR-sensor-based speed detection system is sufficient for the requirements of the ISRW.

### Experimental animals

Adult male Wistar rats (weighing 226–248 g) were obtained from the Animal Resource Center of the National Science Council, Taiwan. They were housed in cages, with four rats per cage, at an ambient temperature of 22 ± 1°C with a 12-h light/dark cycle. Pellet rat chow and tap water were available ad libitum. The experimental protocol was approved by the Animal Ethics Committee of the Chi Mei Medical Center (Tainan, Taiwan), and it was in conformance with the guidelines of the National Science Council of Taiwan.

### Exercise training protocol

Test rats were randomly assigned to one of the following five groups: sham (*n* = 9), control (*n* = 9), treadmill (*n* = 9), MRW (*n* = 9), and ISRW (*n* = 9). Rats in the with-exercise groups (treadmill, MRW, and ISRW) were placed on a treadmill (Exer-6M Treadmill, Columbus Instruments, USA), MRW, or ISRW, as appropriate, for three days of learning mode (human controlled) training to achieve the initial required exercise intensity of 20 m/min for 30 min. Immediately thereafter, the with-exercise groups were subjected to automatic mode training that lasted five days a week for three weeks. Both ISRW and MRW automatic training methods followed the adaptive training model described earlier, whereas for treadmill training, a conventional method in which experimenters manually raised the running speed from slow to fast (0 to 30 m/min) was used. The ISRW and MRW automatic training methods involved running a total of 30 min at 20 m/min in the first week, 30 min at 30 m/min in the second week, and 60 min at 30 m/min in the third week. The work rate of the rats in this training protocol was approximately 70%–75% of their maximum oxygen consumption [39]. The ambient temperature was controlled at 25 ± 1°C. The animals in the control and sham groups did not receive any exercise training but were allowed to run freely in their cages for three weeks.

### MCAo and cerebral infarction assessment

After three weeks of exercise training, transient MCAo (the stroke model) was induced and maintained for 60 min in all rats by using the intraluminal vascular occlusion method [40]. Rats were anesthetized and maintained with 1–3% halothane in 70% N_2_O and 30% O_2_ with a facemask. Briefly, we exposed the left common carotid artery (CCA), external carotid artery (ECA), and internal carotid artery (ICA) by making a midline incision. The CCA and ECA portions were then tied with a white thread. A filament (4–0 nylon suture with a blunted tip 0.37 mm in diameter, Doccol Corporation, USA) coated with poly-L-lysine was inserted into the left CCA through an arteriotomy. Next, the suture direction was changed toward the ICA, and the suture was extended to occlude the MCA. Brain ischemia was measured using a laser Doppler perfusion monitoring instrument (OxyLite 2000, Oxford Optronix, Oxford, UK). During MCAo, the cerebral blood flow decreased from the resting levels (100%) to 40 ± 6% (in the control group), 42 ± 4% (in the treadmill group), 40 ± 4% (in the MRW group), and 42 ± 6% (in the ISRW group). Reperfusion was established by withdrawing the filament under anesthesia at the end of the seventh day. The reliability and effectiveness of this model in inducing stroke was guaranteed by using poly-L-lysine-coated intraluminal sutures, a technique that yields consistently large infarcts and greatly reduces interanimal variability [41]. In the sham group, both ECA and ICA were isolated but not ligated. Infarct volume analysis was performed after seven days of behavior evaluation using the mNSS and inclined plane device test, which are discussed in the following section. The brains were carefully removed and dissected into 2-mm coronal sections by using a vibratome. To measure ischemic change, brain slices were stained in a solution containing 2,3,5-triphenyltetrazolium chloride (TTC) in normal saline at 37°C for 30 min, as detailed previously [42][43]. We also corrected the distortion of the infarct volume caused by brain edema by using the method of Lin [44]. We used an indirect method to calculate the infarct volumes: infarct volume percentage = (area of the contralateral hemisphere − area of the normal region in the ipsilateral hemisphere) divided by the area of the contralateral hemisphere × 100%.

### Modified neurological severity score

Functional outcome was evaluated using the mNSS [37]. All the rats could perform the tests for seven days after surgery. The rats were evaluated using several standard tests, such as lifting the rat by the tail, placing the rat on the floor, and beam balance walking. All the test scores were added to the mNSS. Neurological deficit was graded on a scale of 0 to 18 (normal and healthy score = 0; maximum deficit score = 18), where one point was given for the inability to perform a test or for the lack of a tested reflex. Thus, a higher score indicated a more severe injury.

### Inclined plane test

An inclined plane device was used to evaluate the motor performance [38] of all rats once a day for seven days after surgery. The inclined plane test assesses the ability of the rat to prevent itself from falling, determining the endurance and strength of the lower limbs; the test is a quantitative, objective, and sensitive method for evaluating motor deficits following cerebral ischemia in rats. Initially, the lower limbs of a rat are allowed to clutch the hook side of a Velcro fabric while the upper limbs remain on the acrylic surface of a testing plane inclined at 25°. The angle of the inclined plane is then increased until the lower limbs are detached from the Velcro and the rat slides down, whereupon, the maximal angle is recorded.

### Statistical analysis

All the data are presented as the mean ± SD. Comparisons between the various groups were performed using one-way analysis of variance (ANOVA) followed by Fisher’s LSD post hoc test. The data were analyzed using Sigma Plot software, with the statistical significance set at *p* <0.05.

## Results

### Percentage of effective exercise activity

**Fig 9:**
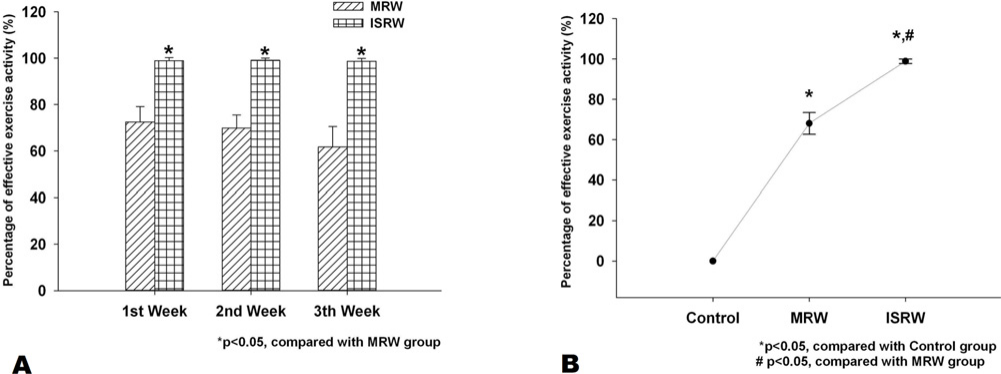
Comparing the percentage of effective exercise activity in the ISRW (*n* = 9) and MRW (*n* = 9) groups. (a) The average effective exercise activity each week. (b) The average effective exercise activity at three weeks.

The four IR LED/detector pairs embedded near the wheel of the proposed ISRW were used to determine the EEA ratio. Fig 9(a) compares the EEA of the MRW and ISRW groups over the three test weeks. The EEA in the ISRW group was higher than that in the MRW group for every week (first week: 98% vs. 75%; second week: 98% vs. 71%; third week: 98% vs. 62%). In other words, the ISRW group almost achieved completely effective (100%) exercise activity. By contrast, the third week training of the MRW group achieved only 62% EEA because the rats could not maintain the running speed and fell down, interrupting the exercise. Consequently, nearly 30% of the training time of the MRW group did not involve continuous and stable running. Fig 9(b) shows the overall average EEA ratio during the three tested weeks in the control, MRW and ISRW groups. The ISRW group achieved 98% EEA, which was higher than the 69.3% EEA in the MRW group, and the 0% of the control group. The experimental results clearly indicate that the ISRW system is much more suitable than the MRW system for exercise training of rats.

### Assessment of neurological damage mNSS

**Fig 10:**
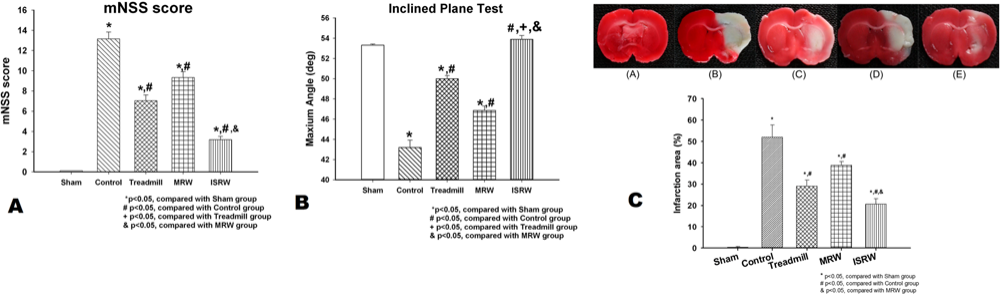
Average mNSS, angle of the lower limb grip test and lesion volume in the five groups (9 rats each group) over a period of 7 days. (a) The average mNSS at 7 days (mean ± SD). (b) The average inclined plane angle at 7 days (mean ± SD). (c) The average lesion volume based on reduced TTC staining (mean ± SD). The top panels of Fig 10(c) depicts a representative photograph of TTC staining for (A) a sham rat, (B) a control group rat, (C) a treadmill group rat, (D) a MRW group rat, and (E) an ISRW rat.

The damaged neurological function after a stroke with exercise preconditioning (treadmill, MRW, and ISRW groups) was assessed using the mNSS. The average mNSS for the five groups seven days after surgery (mean ± SD) are presented in Fig 10(a). The rats in the sham group exhibited no neurological deficits, while the control group exhibited the highest average mNSS (moderate to heavy damage). Relative to the control group, the mNSS of the ISRW group was significantly lower (minor to moderate damage, *p* <0.05). Moreover, the average mNSS of the ISRW group was lower than that of the other exercise preconditioned groups (treadmill and MRW), indicating that the ISRW system provides more apparent and effective neuroprotection from stroke compared with the other commercially available animal motion platforms. These results indicate that forced exercise preconditioning on the ISRW is highly effective in improving recovery from stroke. The scores for the sham group were significantly lower than those for the exercise preconditioning groups (*p* <0.05), indicating no apparent recovery of nerve damage over seven days.

### Motor function

The inclined plane was used to quantitatively measure the motor performance for seven days after surgery. Fig 10(b) shows the average angle obtained from the lower limb grip test for each group over the seven days of testing (mean ± SD). The inclined plane angle of the control group was the lowest, being significantly lower than that of the sham group on each of the seven days. The inclined plane angles of the exercising groups were significantly higher than the control group over the seven days (Fig 10(b); *p* < 0.05), demonstrating the benefit of exercise preconditioning. More importantly, the inclined plane angle of the ISRW group was the highest among the exercise preconditioned groups and was close to that of the sham group, indicating that the recovery of the motor function in the ISRW group was significantly better than that in the other exercise groups (traditional treadmill and MRW).

### TTC staining for evaluation of infarct volume

TTC-stained sections were used to determine the infarct volume in ischemic rats [45]. The normal brain stained red, while the cerebral infarct regions in the brains of the model rats with MCAo showed reduced staining, clearly delineating infarct areas. The experimental results indicated that the infarct size in the control group (291 mm^3^ ± 20%, *p* <0.05) was significantly larger than that in the sham group after seven days of reperfusion (Fig 10(c)). The infarct size in the ISRW (22 mm^3^ ± 4%, *p* <0.05) group was the lowest among all the with-exercise groups and was significantly lower than that in the control group (Fig 10(c)). These data indicate that endurance exercise training with the ISRW reduced the infarct volume more effectively than endurance exercise training with the current commercially available platforms.

### Relationship between EEA and infarct volume

**Fig 11:**
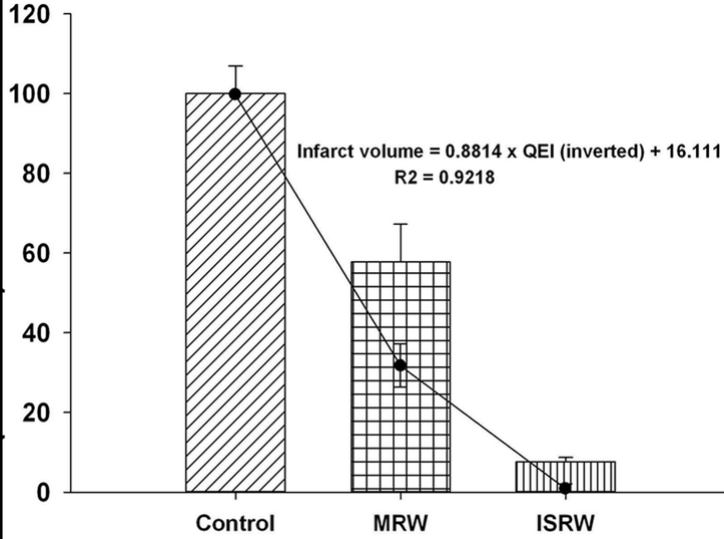
A comparison of the effective exercise activity results with the infarct volumes (Fig 9(b) was inverted and super-imposed on Fig 10(c)).

This study proposed a scientific method to quantify the EEA required for preventing a stroke. An ischemic stroke model was used for validating the effectiveness of this method. When the data in Fig 9(b) are inverted and the data in Figs 9(b) and 10(c) are normalized onto the same scale, a 92% correlative trend between *QEI*_*ISRW*_ and the infarct volume is observed (Fig 11). The 98% EEA in the ISRW group corresponds to the smallest infarct volume. The results show a high correlation between the EEA and the level of brain damage. Therefore, *QEI*_*ISRW*_ of the EEA can be used as an objective noninvasive assessment parameter for clinical experiments on animal exercise.

## Discussion

### Quantitative effectiveness indicator

This study presented an ISRW exercise platform equipped with an infrared system that can detect the position of rodents. The ISRW system allows then quantification and accurate analysis of the influence of effective exercise training in ischemic stroke animal models. More importantly, this study presented a noninvasive IR-sensor-based quantitative effectiveness indicator (*QEI*_*ISRW*_) of effective exercise. The parameter *QEI*_*ISRW*_ was found to correlate highly but inversely with the infarct volume. While training the MRW group, the rats were forced to run at 20 m/min or 30 m/min on the MRW runway. At such times, the rats sometimes clutched the bars and fell, which resulted in physical injury. A similar observation was made in previous experiments using commercially available MRWs [26]. MRW systems are used predominantly in low to medium intensity training experiments because rats reluctantly sustain high-intensity running on these systems [26][33][34]. This is also why the literature has no neurophysiological experiments comparing treadmills with MRWs for the same training intensity. However, the proposed ISRW system successfully allows a comparison between a treadmill and an ISRW system for the same high-intensity exercise. Importantly, this study showed that the ISRW system provides more effective exercise training for protection against and recovery from ischemic stroke in animal models compared with a treadmill and an MRW at the same training intensity. Furthermore, the acrylic runway with the PVC track resolved the grabbing/stumbling problem that characterized previous high-intensity exercise studies, preventing frequent interruptions, which were observed in the traditional MRW system. Moreover, the larger space and flatter runway of the ISRW design allows rats to run more effectively and comfortably, making the design more suitable for high-speed running compared with commercially available MRW. Different types of running exercises (forced and voluntary) affect the neurophysiological performance differently [8][28][29]. In rats, forced exercise effectively induces neuroprotection with regard to stroke [8][28]. Voluntary exercise does not induce neuroprotection for stroke, but voluntary activity leads to more prominent plastic changes in the hippocampal formation of rats compared with forced exercise [30]. The current study focused on a performance comparison of forced exercise platforms.

### Comparison of various animal motion platforms

Table 1 presents a comparison of existing animal locomotion platforms (treadmills, free wheels, commercially available MRW, and the proposed ISRW). The main advantages of the ISRW system are its capability to facilitate various intensities (low to high) of exercise training and to calculate *QEI*_*ISRW*_ of the EEA. Moreover, the psychological pressure exerted on animals is lower for the ISRW system compared with treadmills. The ISRW design overcomes the difficulties faced in high-intensity training on commercially available MRWs and treadmills, and allows the effects of high-intensity exercise on the infarct volume in cerebral ischemic stroke animals to be verified without interference from physiological stress or exercise interruptions.

**Table 1:** Comparison of various animal motion platforms.

Function	Treadmill	Free running wheel	MRW	ISRW (this study)
Exercise training method	Forced (electrical shock)	Voluntary	Forced (motorized from center)	Forced (motorized from side)
Training intensity	Low, medium, high	Low, medium, high	Low, medium	Low, medium, high
Number of simultaneously training animals	Plurality	Single	Single	Single
Training time control	Yes	No	Yes	Yes
Automatic adaptive acceleration training	No	No	No	Yes
Runway structure	Rubber belt	Bars	Bars	Textured rubber belt
Running position detection	No	No	No	Yes
Effective exercise assessment	No	No	No	Yes
Psychological stress	High	None	Low	Low
Cost	High	Low	Medium	Low to medium

The experimental results indicate that the exercising groups experienced more neuroprotective benefits compared with the control group, which was expected. In addition, the ISRW platform had a more significant effect than the other platforms (traditional treadmill and MRW). The experimental mNSS, inclined plane test results, and infarct volume results for the ISRW group were significantly better (Fig 10; *p* <0.05) compared with those for the control group. Moreover, the mNSS, inclined plane test results, and infarct volume were better for the ISRW group compared with the MRW and treadmill groups, indicating that the ISRW provides more effective exercise training than the currently used animal motion platforms. The commercially available forced exercise platforms (treadmill and MRW) introduce certain factors that affect exercise. The protection against ischemic stroke provided by a treadmill is partly a result of the use of an electrical stimulus [28]. However, clinical researchers have provided evidence for electric shocks in treadmills evoking systemic stress, which may lead to negative physiological effects, especially in male animals [46][47]. Such experimental data are subject to different interpretations with regard to what constitutes “mental stress” versus “exercise adaptation” [48][49]. Evidence has confirmed that a treadmill generates more apparent psychological pressure than free or forced running wheels [8][28–31].

### Improved factors over other platforms

The ISRW shows better performance compared with the treadmill. One reason for this improved performance is the diminished psychological pressure resulting from the lack of electrical shocks. Electric shocks not only induce significant psychological fear in rats [8][28][30] but also interrupt the rhythm of the exercise, thereby influencing the EEA. Although the EEA of the treadmill is not defined, it can be observed that the exercise in periods in which the exercising rat falls into the treadmill’s shock region cannot be regarded as EEA in experiments. This is not the main subject of this study. However, when the EEA is reduced, the degree of neurophysiological protection decreases. These negative factors reduce the beneficial effects of exercising on a treadmill. The EEA is lower on an MRW compared with that on an ISRW, and the corresponding infarct volume after MRW training is greater than that after ISRW training. In other words, a shorter training time on the ISRW can achieve benefits similar to those of the MRW. The relatively small and restricted size of the MRW’s running track results in a higher probability of rats stumbling at high training speeds. Consequently, the EEA of rats on an MRW is reduced, which adversely affects the efficacy of neurophysiological protection. Although, the PVC belt of the VRW fabricated in the current study allows rats to run at 30 m/min on the wheel, the small dimensions of the wheel appear to be one of the key factors influencing the degree of EEA. Both the cost and capability of the driving motor would increase with use of a bigger wheel. These factors must be considered for the design tradeoffs during the design of the MRW.

The presented ISRW platform provides *QEI*_*ISRW*_, a noninvasive quantitative effectiveness indicator of the EEA. This parameter follows the same trend as that of the infarct volume, which provides the most accurate measure of the extent of stroke injury. Therefore, *QEI*_*ISRW*_ can provide a convenient reference in clinical animal experiments. The experimental results of the current study indicate that effective exercise correlates with lower infarct volume and better neurological function. Previous studies controlled only the intensity and duration of exercise for evaluating the efficacy of clinical neurophysiology [11][21–23]. However, ineffective exercise sometimes occurs during the training process, for example, when a rat stumbles on a running wheel or when electric shocks in a treadmill interrupt the pace of exercise. Currently, IEA is mostly ignored. This study, however, shows that the EEA is one of the primary mediators of neurophysiological protection. Therefore, the ISRW system not only provides a more efficient exercise training platform that facilitates effective neurophysiological protection but also provides *QEI*_*ISRW*,_ which quantifies the EEA.

## Conclusion

This paper presents an ISRW that is a more effective animal exercise training platform compared with the traditional treadmill and the traditional motorized running wheel (MRW). The use of the ISRW leads to a small infarct volume and large improvement of the neurological function after a stroke. Traditional treadmills apply electric shocks to rats to make them run, a factor known to lead to many negative physiological consequences. The proposed ISRW system eliminates the need for electric shocks. The traditional MRW generally discourages rats from running at a high speed. We fabricated a traditional-sized MRW with PVC flooring, allowing rats to run at high speed.

The ISRW system was implemented and verified, and the two motion platforms (the traditional treadmill and the MRW) were also tested in this research. To date, only the training speed and total exercise time have been controlled for evaluating the neuroprotective effects of animal exercise platforms. The amount of effective exercise during training was not considered. This study demonstrated that the duration of the EEA is one of leading factors affecting the degree of neuroprotection. The highest EEA of 98% in the ISRW group corresponds to the smallest infarct volume among the exercise groups. The parameter *QEI*_*ISRW*_ showed a 92% correlation with the inverse of the infarct volume, indicating that the EEA affects the extent of neuroprotection. Clinical test results confirmed that the ISRW platform achieved a more obvious and appreciable neuroprotective effect compared with conventional methods, proving it to be a superior choice for stroke prevention studies.
